# A Retrospective Study of Risk Factors and Outcomes in the Surgical Management of Slipped Capital Femoral Epiphysis

**DOI:** 10.5435/JAAOSGlobal-D-21-00135

**Published:** 2022-07-06

**Authors:** Winston Jin, Sarah Farrell, Eva Habib, Ash Sandhu, Jeffrey N. Bone, Emily Schaeffer, Kishore Mulpuri

**Affiliations:** From the Department of Orthopaedic Surgery, BC Children's Hospital, Vancouver, BC, Canada (Jin, Habib, Dr. Schaeffer, and Dr. Mulpuri); the Children's Hospital Queensland, South Brisbane, Australia (Farrell); the Department of Orthopaedics, Faculty of Medicine, University of British Columbia, Vancouver, BC (Dr. Schaeffer, and Dr. Mulpuri); and the BC Children's Research Institute, University of British Columbia, Vancouver BC (Sandhu, and Bone).

## Abstract

**Purpose:**

Slipped capital femoral epiphysis is commonly treated with in situ pinning (ISP) and more recently the modified Dunn procedure (MDP). This study retrospectively examines the preoperative risk factors and postoperative complications of patients treated with either ISP or MDP over a 12-year period.

**Methods:**

A single-center, retrospective review was conducted on patients diagnosed and surgically treated with slipped capital femoral epiphysis from 2004 to 2016. Patients must have had preoperative imaging and a minimum of 6 months of clinical follow-up. Six preoperative demographic data (age, sex, intensity of symptoms, stability, trauma, and severity of slip), surgical details, and treatment outcomes were collected. Descriptive statistics were used to identify pertinent preoperative risk factors and postoperative complications in each treatment group.

**Results:**

A total of 129 hips in 98 patients were treated (118 with ISP and 11 with MDP). Complications developed in 12 hips. Six hips developed osteonecrosis, two hips developed osteonecrosis and chondrolysis, two hips developed osteonecrosis and slip progression, and two hips developed slip progression only. Four of the 11 hips (36.4%) treated with MDP developed complications; 8 of the 118 hips (6.8%) treated with ISP developed complications.

**Discussion:**

Complications developed in 9.3% of hips treated with ISP or MDP, with a higher rate of complications observed in the MDP group compared with the ISP group. This study is limited by the small sample size of the cohort and the disproportion in the number of cases in each treatment group. A multicenter study with larger sample sizes will be required to confirm these findings.

Slipped capital femoral epiphysis (SCFE) is one of the most common disorders of the adolescent hip, and it is characterized by the displacement of the capital femoral epiphysis from the femoral neck along the physeal plate.^1,2^ Several classification systems are used during the initial diagnosis of SCFE to categorize the extent of the disease. The intensity of SCFE can be classified based on the length of time symptoms have persisted as acute (<3 weeks), chronic (>3 weeks), or acute-on-chronic.^3^ Physeal stability is also assessed during diagnosis: a SCFE is considered stable if a patient is able to weight bear and unstable if a patient is unable to weight bear, even with the aid of crutches.^4^ Radiographical examination with anteroposterior and frog lateral pelvis radiographs is used to image the hip joint and visually determine the presence of a slip. From the frog lateral pelvis radiographs, the Southwick Slip Angle Classification is used to categorize the severity of the slip. Based on the femoral epiphyseal-diaphyseal angle difference, aSCFE is considered mild when the Southwick angle is <30°, moderate when the Southwick angle is between 30° and 50°, and severe when the Southwick angle is >50°.^5^

Once diagnosed, patients with SCFE are usually treated with the well-established and commonly-used method of in situ pinning (ISP) with one cannulated screw to stabilize the hip as soon as possible.^6^ Despite the relatively uncomplicated nature of SCFE's pathology and treatment, many long-term and irreversible complications can develop from SCFE that lead to early disability and possible hip reconstruction surgery.^7^ Postoperative complications include osteonecrosis of the femoral head, chondrolysis, and slip progression, which are all major concerns associated with SCFE.^6^

Osteonecrosis of the femoral head is the most serious complication of SCFE and is involved mostly in unstable slips, complicating 24% to 47% of unstable cases.^4,8^ Among those with an unstable slip, younger age and shorter duration of prodromal symptoms at presentation are associated with the development of osteonecrosis.^9,10,11,12^ SCFE patients with osteonecrosis develop early disability of the hip and the need for total hip arthroplasty within the first 10 years after the slip, as opposed to SCFE patients without osteonecrosis who will undergo total hip arthroplasty on average 23.6 years after the slip because of postslip degenerative arthritis.^13^ Osteonecrosis is also the most common cause for hip arthroplasty in patients with SCFE compared with other complications.^13^

Chondrolysis is characterized as ongoing articular cartilage damage of the hip joint, causing unresolved pain and stiffness of the hip after ISP.^6,7^ Patients with chondrolysis may present with additional restriction of internal rotation of the hip and a worsening limp, as well as persistent pain located at the hip joint or referred to the ipsilateral anterior thigh or knee.^20,21^ The incidence of chondrolysis is 5 to 7%, and the etiology for this complication has not been clearly identified.^6,7^ In addition to complications inherent to SCFE, one of the complications related to the implants used in ISP is slip progression, which usually occurs when there is implant failure or when there is continuing femoral neck growth that causes the epiphysis to outgrow the screw implant.^6,7^ As a result, the epiphysis is no longer stabilized by the screw, leading to further slipping on the femoral neck.^6,7,22^

Femoroacetabular impingement (FAI) is a common development from SCFE treatment, affecting from 32% to 90% of all SCFE patients, including those with a milder form of the disease.^6,7,17,18^ FAI occurs when the deformed femoral neck pushes against the acetabular labrum and the acetabular articular cartilage during flexion and internal rotation of the affected hip, causing pain and stiffness.^7^ Studies suggest that, regardless of the severity of the slip, 80% to 90% of treated slips will eventually present with labral and acetabular cartilage lesions.^6,18,19,20,21,22^ Owing to the high prevalence of FAI in the long run, it could be regarded not as a complication but as the end point of the natural history of SCFE, regardless of treatment.^6,7^ Thus, FAI was not considered a complication in this study.

More recently, open surgeries such as the modified Dunn procedure (MDP) have been increasingly adopted as a form of treatment for SCFE.^12^ The goal of treating SCFE with MDP instead of ISP was to decrease the risk of osteonecrosis, especially in patients with unstable slips, by preserving the blood supply to the femoral head.^12^ MDP achieves this with a surgical dislocation of the epiphysis, an osteotomy of the greater trochanter, and finally the realignment and internal fixation of the capital epiphysis.^23^ However, MDP is a more complicated procedure and more demanding for surgeon expertise and skill.^24,25,26,27,28,29,30,31,32^ Clinical outcomes for MDP vary, with some studies reporting favorable postoperative outcomes while others showing higher rates of osteonecrosis.^12,33,34,35,36,37,38,39,40^ Research is ongoing to determine parameters to aid in selecting the most appropriate treatment and determining which treatment is least likely to result in postoperative complications.^24,25,30–32^ The goal of this study was to examine the preoperative risk factors and postoperative complications in patients treated with either ISP or MDP using data from a pediatric tertiary care center. A comparison between patient outcomes of the two treatment methods is conducted to compare the rate of complications of these treatment groups.

## Methods

A single-center, retrospective radiographic and chart review was conducted on patients who have been surgically treated for SCFE from 2004 to 2016 at a pediatric tertiary care center. To be included, patients must have been younger than 18 years at presentation, diagnosed with SCFE, and treated surgically by ISP or MDP. Treatment was decided based on clinical judgment and experience of the surgeon in conjunction with discussion about the preferences and values of the individual patient. Patients must have had preoperative imaging and a minimum 6 months of postoperative radiographic and clinical follow-up.

Demographic data such as age and sex were recorded. Clinical and radiographical diagnosis and preoperative risk factors were also collected, including the side of the affected hip (left, right, or bilateral), intensity of symptoms (acute, acute-on-chronic, or chronic), physeal stability (stable or unstable), severity based on Southwick angle (mild, moderate, or severe), and trauma before presentation (trauma or no trauma).

Treatment and surgical details (ISP or MDP), as well as postoperative clinical and radiographical outcomes such as the development of complications, were collected. Osteonecrosis of the femoral head and slip progression were identified retrospectively from both clinical and radiographical records, whereas chondrolysis was identified from descriptions in the clinical records only. Study data were collected and managed using REDCap electronic data capture. REDCap (Research Electronic Data Capture) is a secure, web-based software platform designed to support data capture for research studies, providing (1) an intuitive interface for validated data capture, (2) audit trails for tracking data manipulation and export procedures, (3) automated export procedures for seamless data downloads to common statistical packages, and (4) procedures for data integration and interoperability with external sources.^41,42^ Each set of patient data was coded with a subject ID before being entered into REDCap and did not contain any patient's name or personal identifying information.

## Results

A total of 130 patients diagnosed with SCFE were identified from surgical databases and clinic lists of the individual participating surgeons at this pediatric tertiary care center. Of these patients, 32 were excluded from this study because they did not meet all the inclusion criteria. Specifically, 16 patients did not have a minimum 6 months of postoperative radiographic and clinical follow-up, nine patients did not have accessible preoperative imaging, two patients did not have accessible preoperative clinical data, and one patient was not treated with either ISP or MDP.

In the 98 eligible patients with SCFE, 67 patients (68.4%) were treated for a unilateral slip only, 7 patients (7.1%) were treated for a unilateral slip and also had their contralateral hip prophylactically pinned with ISP, 11 patients (11.2%) were initially treated for a unilateral slip but developed and treated for a contralateral slip later on, and 13 patients (13.3%) were treated for bilateral slips. Of a total of 129 hips that underwent surgical treatment, 118 hips (91.5%) were treated with ISP and 11 hips (8.5%) with MDP. Six preoperative risk factors (sex, age, intensity, stability, severity, and trauma) and the number of complications were specified a priori and recorded for descriptive analysis, as presented in Table 1.

**Table 1:** Demographic Data, Preoperative Risk Factors, and Outcome by Procedure

Total (ISP + MDP)	ISP	MDP
Sex		
Male: 58 (59.2%)	Male: 53 (60.9%)	Male: 5 (45.5%)
Female: 40 (40.8%)	Female: 34 (39.1%)	Female: 6 (54.5%)
Total patients: 98	Total patients: 87	Total patients: 11
Age (yrs)		
Min.: 8.10	Min.: 8.10	Min.: 9.0
Median: 12.40	Median: 12.40	Median: 11.9
Mean: 12.28	Mean: 12.36	Mean: 12.02
Max.: 17.60	Max.: 17.60	Max.: 14.0
Intensity		
Acute: 16 (12.4%)	Acute: 16 (13.6%)	Acute: 0 (0.0%)
Chronic: 74 (57.4%)	Chronic: 71 (60.1%)	Chronic: 3 (27.3%)
Acute-on-chronic: 39 (30.2%)	Acute-on-chronic: 31 (26.3%)	Acute-on-chronic: 8 (72.7%)
Total hips: 129	Total hips: 118	Total hips: 11
Stability		
Unstable: 33 (25.6%)	Unstable: 28 (23.7%)	Unstable: 5 (45.5%)
Stable: 96 (74.4%)	Stable: 90 (76.3%)	Stable: 6 (54.5%)
Severity		
Severe: 39 (30.2%)	Severe: 31 (26.3%)	Severe: 8 (72.7%)
Moderate: 46 (35.7%)	Moderate: 45 (38.1%)	Moderate: 1 (9.1%)
Mild: 25 (19.4%)	Mild: 25 (21.2%)	Mild: 0
NA: 19 (14.7%)	NA: 17 (14.4%)	NA: 2 (18.2%)
Trauma		
Yes: 37 (28.7%)	Yes: 32 (27.1%)	Yes: 5 (45.5%)
No: 92 (71.3)	No: 86 (72.9%)	No: 6 (54.5%)
Complication		
No: 117 (90.7%)	No: 110 (93.2%)	No: 7 (63.6%)
Yes: 12 (9.3%)	Yes: 8 (6.8%)	Yes: 4 (36.4%)

ISP = in situ pinning, MDP = modified Dunn procedure

Complications developed in 12 of the 129 hips (9.3%), six of which developed osteonecrosis only, two developed osteonecrosis and chondrolysis, two developed osteonecrosis and slip progression, and two developed slip progression only. Of the 11 hips treated with MDP, four hips (36.4%) developed complications while eight hips (6.8%) in the 118 hips treated with ISP developed complications. The number and type of complications in each treatment group are summarized in Table 2. In addition, 7 of the 11 hips (63.6%) treated with MDP had revision surgeries: 4 of the seven (57.1%) had revision surgeries to correct their postoperative complication as described, 1 (14.3%) had a broken implant removal and repair procedure, and 2 (28.6%) had screw removals because of the screw backing out. Comparatively, 18 of the 118 hips (15.2%) treated with ISP had revision surgeries: 8 of the 18 hips (44.4%) were treatment for their postoperative complication as mentioned, 7 (38.9%) were screw revision/removal for persistent pain, 2 (11.1%) were screw removal from penetration into joint space, and 1 (5.6%) was a wound infection débridement.

**Table 2:** Number of Cases by Complication in ISP, MDP, and Total

Total (ISP + MDP): 129 cases	Complication	Osteonecrosis	Osteonecrosis + chondrolysis	Osteonecrosis + slip progression	Slip progression
	Cases	6 (4.7%)	2 (1.6%)	2 (1.6%)	2 (1.6%)
ISP: 118 cases	Complication	Osteonecrosis	Osteonecrosis + chondrolysis	Osteonecrosis + slip progression	Slip progression
	Cases	4 (3.4%)	1 (0.8%)	2 (1.7%)	1 (0.8%)
MDP: 11 cases	Complication	Osteonecrosis	Osteonecrosis + chondrolysis	Osteonecrosis + slip progression	Slip progression
	Cases	2 (18.2%)	1 (9.1%)	0	1 (9.1%)

ISP = in situ pinning, MDP = modified Dunn procedure

## Discussion

Although SCFE is one of the most common hip conditions in adolescents, the overall incidence is relatively low with reports ranging from 0.33 to 24.58 of 100,000 children aged 8 to 15 years depending on the geographic location.^43^ With the possibly large spectrum of risk factors, varying degrees of slip severity, and the multiple treatment options available, this low incidence has limited the ability to comparatively assess outcomes to a meaningful capacity. SCFE is commonly treated with stabilization of the epiphysis through ISP.^6,7,30^ ISP typically results in good clinical outcomes for stable slips; however, it has been found that with unstable or severe stable slips, there can often be future complications that lead to FAI and articular cartilage damage leading to the development of osteoarthritis.^10,24,25,30^ As well, there remains the concern of the high incidence of osteonecrosis, particularly in unstable slips.^6,7,8,9,10,11,12^ Consequently, MDP was developed for moderate-to-severe slips to combat this issue.^29,30^ MDP involves a surgical hip dislocation, creation of a retinacular flap, open reduction of the epiphysis, and internal fixation of the slip to surgically correct the pathoanatomy, avoiding future cartilage damage.^23,25,27^

Recent studies have shown that MDP leads to better clinical and radiographic improvement than ISP when treating stable SCFE.^34,38^ A 2015 study by Novais et al showed that MDP led to better deformity correction, higher rates of good and excellent Heyman and Herndon clinical outcome, and a lower revision surgery rate compared with ISP for treatment for severe stable SCFE.^34,44^ A retrospective 2019 study by Ebert et al^38^ found similar improvements in morphologic features of the femur in patients with severe stable chronic or acute-on-chronic SCFE treated with MDP. However, there is evidence that complication rates, specifically both osteonecrosis and postoperative hip instability rates, are higher in stable SCFE compared with unstable SCFE when treated with MDP.^36,37^ A retrospective review by Davis and colleagues^36^ in 2019 found that 29.4% of the patients with stable SCFE treated with MDP developed osteonecrosis and 6% developed osteonecrosis in the unstable group. Souder et al,^39^ in 2014, found a 20% rate of osteonecrosis in patients with stable SCFE treated with MDP compared with 0% in patients treated with ISP, whereas they found no difference in the osteonecrosis rate in patients with unstable SCFE treated with either MDP or ISP. Thus, the author suggests treating chronic, stable SCFE with ISP and treating unstable SCFE with either MDP or ISP because of high complications rates in this patient population in general.^39^ In addition, Upasani et al,^40^ in 2014, first described the complication of postoperative hip instability or dislocation in SCFE patients treated with MDP, which occurred in 5% of their patients in this population. Although the outcome is promising for correcting the anatomic deformity of SCFE and reducing osteoarthritis, MDP may pose a greater concern for inducing osteonecrosis of the femoral head than is found with ISP, particularly in stable hips.^27,35,36^ Research is ongoing to determine how to select the ideal treatment for a patient; however, one study found that experienced surgeons prefer MDP, and another study showed that surgeon experience is directly associated with patient outcomes.^26,40^

Although the literature points toward stability of the hip as a possible factor when choosing between treatment methods, we were interested in whether there are other preoperative conditions that could lead to postoperative complications in patients treated with either ISP or MDP, thus pointing toward more clear indications for choosing one method of treatment versus the other. The results from our study were consistent with existing literature because we observed higher complication rates in the MDP treatment group (36.4%) compared with the ISP treatment group (6.8%). In addition, revision surgery rates were higher in hips treated with MDP (63.6%) compared with ISP (15.25%). However, a notable portion (72.7%) of the hips treated with MDP were severe slips, which might have required revision surgeries, regardless of the treatment method. Our study is limited by the small overall sample size of SCFE cases and, in turn, few postoperative complications. In addition, the MDP treatment group had markedly fewer patients compared with the ISP treatment group. A multicentre review would provide the volume of patients required to more accurately evaluate preoperative risk factors and treatment complication rates in the management of this condition. A strength of our study is the wide date range that includes more experienced surgeons and new ones, thus providing an accurate representation of surgical outcomes across different skill levels. Our evaluation contributes to the identification of optimal treatment methods (MDP versus ISP) and six identified risk factors (age, sex, intensity of symptoms, stability, trauma, and severity of slip) for complications in the management of SCFE; thus, it can aid in the development of recommendations for treatment based on different forms of the disease.

Our study has a number of limitations. First, there is the possibility of a selection bias because only a small number of all patients with SCFE were treated with MDP, whereas the majority was treated with ISP. Second, the identification of chondrolysis was made based on descriptions from clinical data from physician charts, and no objective guidelines for radiographical evidence to identify chondrolysis were available to be used in this process. This subjects the identification of these complications to recall bias, affecting the accuracy of the data. Third, owing to the retrospective nature of the study, 32 patients were excluded from the study because of incomplete data or loss of follow-up. Fourth, other potentially relevant demographic data, such as patients' body mass index, hormonal profiles, and evidence of decreased bone density, were not available and were beyond the scope of this study. These limitations point toward a need for a more rigorous prospective study with a comprehensive patient log and the initiation of a study that records patients' conditions before surgery and their long-term outcomes across multiple participating centers, which may find differences we did not and provide valuable insight into the treatment and management of SCFE.

As we work toward improving the outcomes for children diagnosed and treated for SCFE and in the process establishing the optimal treatment method for the disease, we must consider the treatment variations and available resources from a global perspective. The results from our project indicate a need for a large-scale study with an increased sample size and diversity of treatment. An international data registry for SCFE such as the Slipped Longitudinal International Prospective Registry is a crucial platform that allows collaboration between hospitals on an international stage. Contribution to and analysis on the Slipped Longitudinal International Prospective Registry will maximize the effect on children with SCFE worldwide.
